# Telemedicine and psoriatic arthritis: best practices and considerations for dermatologists and rheumatologists

**DOI:** 10.1007/s10067-022-06077-3

**Published:** 2022-01-26

**Authors:** Alice B. Gottlieb, Alvin F. Wells, Joseph F. Merola

**Affiliations:** 1 Icahn School of Medicine at Mount Sinai, 10 Union Square East, New York, NY USA; 2 Aurora Rheumatology and Immunotherapy Center, Franklin, WI USA; 3 Brigham and Women’s Hospital, Harvard Medical School, Boston, MA USA

**Keywords:** Clinical practice, Dermatology, Psoriatic arthritis, Rheumatology, Telemedicine

## Abstract

Telemedicine encompasses a variety of modalities that allow for the remote assessment and treatment of patients. The technologies, services, and tools available for telemedicine in the USA are increasingly becoming an integral part of the healthcare system to bridge the gaps in care that can arise from geographic and/or socioeconomic obstacles and provider shortages. Telemedicine can be applied to a spectrum of clinical areas, including rheumatic diseases. Psoriatic arthritis (PsA) is a chronic, inflammatory, multisystem disease with predominately skin and joint manifestations. PsA is often misdiagnosed and/or undiagnosed, which can lead to worse patient outcomes, including irreversible joint erosion and damage. The difficulties in diagnosing and managing PsA are confounded by the emergence and increased use of telemedicine because of the COVID-19 pandemic. Telemedicine presents the opportunity to increase access to healthcare by rheumatologists and dermatologists to improve training and education regarding PsA and to decrease time attributed to office visits associated with PsA. However, challenges in diagnosing PsA without a thorough in-person physical examination by a trained rheumatologist or dermatologist exist. We provide an overview of the ways telemedicine can be incorporated into clinical care and optimized for patients with PsA; characteristic clinical features of PsA, with a focus on skin and joint signs and symptoms; screening tools to be used in routine clinical care; assessments that can be used to evaluate quality of life, functional ability, and disease activity in PsA; and resources and recommendations for the development of future telemedicine use in rheumatology and dermatology.

**Table:** 

**Key Points** • *Patients with psoriatic arthritis (PsA) are often misdiagnosed and/or undiagnosed.* • *Telemedicine can improve access to healthcare by rheumatologists and dermatologists.* • *Telemedicine can be incorporated into clinical care and optimized for managing PsA.*

**Key Points**

• *Patients with psoriatic arthritis (PsA) are often misdiagnosed and/or undiagnosed.*

• *Telemedicine can improve access to healthcare by rheumatologists and dermatologists.*

• *Telemedicine can be incorporated into clinical care and optimized for managing PsA.*

## Telemedicine for diagnosis and disease management of psoriatic arthritis

Telemedicine refers to the exchange of medical information through electronic communication methods with the purpose of improving patient health [1]. Technology, services, and tools used in telemedicine are increasingly becoming a vital part of the healthcare system in the USA, especially with the emergence of COVID-19. Telemedicine allows for more patients to maintain ongoing appointments, facilitates triage and screening to assess the need for in-person visits, supports communication for shared decision-making to monitor disease maintenance and changes in therapy, and can prevent discontinuation of long-term treatments that may lapse without physician oversight, all while ensuring social distancing to avoid the risk of exposure to the virus.

  Telemedicine can also be applied beyond the context of a pandemic to provide healthcare services within or outside of normal clinic hours, to fill gaps in care that may result from provider shortages, and to decrease travel and cost burdens for office visits, especially in rural areas [1]. Many benefits and challenges should be considered before incorporating telemedicine into routine clinical settings (Table 1) [1–9].

**Table 1:** Benefits and challenges of telemedicine in rheumatology and dermatology

Benefits		Challenges	
Increased access to care	• Patients can be assessed by rheumatologists and dermatologists sooner -Patients can be screened to determine whether an in-person visit is necessary - Patients could be diagnosed and begin treatment faster • Patients can maintain social distancing and prevent the risk of exposure to the COVID-19 • Rapid follow-up for test results feedback • Access to healthcare outside of normal clinic hours • Patients in remote/rural areas have more opportunities to see specialists • Real-time access to electronic screening tools and PRO measures	Financial barriers	• Lack of funding for telemedicine services and tools can be an obstacle to widespread use • Increased costs can be accrued with the need for training to adapt to telemedicine (billing, implementation of e-tools, etc.) • Patient financial means may limit access to reliable internet and tools to ensure quality images and/or video
Increased cost-effectiveness and decreased time spent	• Patients have decreased travel time and expenses with virtual appointments • Healthcare providers who may travel to remote/rural clinics will avoid travel time and expenses • Patients with more severe disease disability can avoid the physical toll of an in-person visit • Decreased in-office visits for patients who need routine monitoring • Reduction in participating site management with the use of mobile devices and virtual visits for clinical trial monitoring and communications	Assurance of quality care	• In the absence of a thorough physical examination, a diagnosis of PsA may be difficult - Particularly with skin assessment, areas hidden from camera may not be evaluated appropriately (e.g., scalp) - Distinction of enthesitis may be difficult for patients to self-assess • A standardized and widely accepted teleconsultation evaluation may be needed to ensure quality diagnosis of PsA • Interpreters may not be available for patients who need communication support • Patients may not feel comfortable with virtual examination of certain body parts (e.g., genitals, breasts) • Diagnostic tests are not readily available with telemedicine
Education and training	• General clinicians can learn more about diagnosis of PsA with the ability to attend telemedicine consultations • Increased training and tutorials regarding how to efficiently diagnosis early PsA can be available • Patients can learn more about their disease and treatment regimen	Clinical and staff support	• Widespread acceptance and advocacy of clinical and support staff is needed for optimal success of telemedicine • Healthcare providers may be cautious of potential HIPAA violations or security breaches • Providers and clinical staff will need to stay up to date on laws and regulations for virtual patient care
Improved communication	• Immediate feedback is possible • Referring clinician has the potential to attend the referral appointment virtually • Increased monitoring of patient status available with electronic PROs can facilitate better shared decision-making between rheumatologists or dermatologists and patients	Technology barriers	• All patients may not have access to high-quality technology to facilitate an optimal evaluation • Patients unfamiliar with and/or who find the use of technology difficult may have trouble setting up virtual tools and/or accessing EMR patient portals, which may leave them unsatisfied with telemedicine
Patient satisfaction	• Good patient satisfaction with telemedicine consultations has been reported		

*EMR* electronic medical record; *HIPAA* Health Insurance Portability and Accountability Act; *PRO* patient-reported outcome; *PsA* psoriatic arthritis

There are generally 3 types of telemedicine interactions to ensure continuity of care: clinician-to-clinician, clinician-to-patient, and patient-to-mobile health technologies [1]. Telemedicine between healthcare providers can be used for consultation with specialists and colleagues through video, email, and/or telephone and supported by transfer of imaging data or clinical results for review. The synchronous use of telemedicine between patients and their healthcare providers can be applied to manage care for chronic conditions, to evaluate response to medications, to assess patient-reported outcomes (PROs) and changes, for mental health evaluations, and for routine follow-up care [10]. Telemedicine allows patients to have a more active role in their diagnosis and disease management through the use of mobile health tools.

Psoriatic arthritis (PsA) is a heterogeneous and complex disease with musculoskeletal and cutaneous involvement, causing potentially severe joint and skin manifestations [11, 12]. PsA is estimated to affect approximately 30% of patients with psoriasis and 0.25% of the total population in the USA [13]. The Group for Research and Assessment of Psoriasis and Psoriatic Arthritis (GRAPPA) identified 6 clinical domains of disease activity as part of its treatment schema: peripheral arthritis, enthesitis, dactylitis, psoriasis, psoriatic nail disease, and axial disease [14]. GRAPPA and the Outcome Measures in Rheumatology Working Group have also developed and updated a core domain set that should be assessed in clinical trials to include the perspectives of patients and clinicians, with the addition of pain, patient’s global assessment (PtGA), physical function, fatigue, health-related quality of life (QoL), and systemic inflammation [15]. A delay in diagnosis and inadequate treatment of PsA can result in an increase in significant irreversible joint damage and disease burden [16–18], and the integration of telemedicine has the potential to improve access to rheumatologists and dermatologists to avoid preventable delays.

## Practical applications for virtual appointments

A workflow protocol for using different types of telemedicine in appointments between a patient and healthcare provider should consist of 3 general stages for effective teleconsultations: previsit screening, day of and during the appointment, and postappointment [10].

Before the appointment, support staff work with patients to provide necessary education to set up and use the technology required for the appointment. The Office for Civil Rights (OCR) at the Department of Health and Human Services (HHS) updated guidance for the use of audio or video communication technology during the pandemic to allow covered healthcare providers to use any non-public facing remote communication product [19]. Outside the context of a public health emergency, when choosing a vendor for virtual visits, the American Medical Association (AMA) has compiled a list of the following key variables to consider to ensure safe and optimal patient care: (1) easy to access, (2) compliant with Health Insurance Portability and Accountability Act of 1996 (HIPAA) and state rules (e.g., willingness to sign a Business Associate Agreement, State Medical Board regulations), (3) has third-party audits, (4) provides options to manage security breaches, (5) user authentication and authorization, (6) transparency on processes used for collected data, (7) in-platform consent capabilities, and (8) dashboard/workflow assimilation [20]. A previsit screening call can be useful to answer questions from the patient, to identify and take photos of key concerns (e.g., skin lesions, dactylitis, nail dystrophy, swollen/tender joints), to ensure appropriate laboratory/imaging tests were completed and shared with the provider, to review expectations for the appointment, and to complete previsit screening and PRO questionnaires. Before seeing patients virtually, providers and support staff should be up to date on current insurance coverage policies to prevent delays in care, because some plans do not cover all aspects of virtual care—including phone calls.

On the day of an appointment, the provider can perform subjective and objective assessments, in combination with asking pointed questions and incorporating clinical tests, to comprehensively evaluate the patient. Objective measures include a provider-led physical examination, during which the clinician visually evaluates affected parts of the body through techniques the patient can self-perform, and through presentation of affected areas of the body that are easily visible. Published frameworks of written and visual guidelines are available that include guidance on how to properly conduct a thorough virtual musculoskeletal physical examination and can be used to assess PsA musculoskeletal involvement; these frameworks include general considerations for telemedicine visits, inspections/observations, patient self-palpations, and range of motion and specific detailed tests to evaluate the shoulder, hip, knee, ankle, and cervical and lumbar spine [21, 22]. Provider-guided physical examinations that focus on the digits and commonly affected entheses can be useful to identify the presence of enthesitis and dactylitis. Furthermore, psoriatic nail and skin disease can be assessed through self-evaluation (e.g., body surface area [BSA] “palm rule” [23] or Self-Administered Psoriasis Area and Severity Index [24]) and/or store-and-forward high-quality images. During a video assessment, directed questioning and examination of areas of the body commonly affected by psoriatic skin disease can aid in an accurate assessment of skin lesions and help the patient identify areas affected that are not readily visible. Images can facilitate identification of presumed psoriatic lesions to assist in a definitive diagnosis and help properly visualize typical nail disease (e.g., pitting, onycholysis, onychomycosis). Additionally, modern electronic tools for self-examination that were designed for clinical trials—such as smartphone sensor tools, smartphone apps, wearable health monitors, bodily fluid diagnostic devices, wearable patches, mobile dolorimeters, and at-home self-examination kits [25–28], while not widely available—can be used as research tools and are in development for virtual care to directly relay accurate results to providers and increase the assessment capabilities available during virtual appointments [29]. Subjective assessment of a patient’s overall health is important to gauge complete therapeutic response and can include PRO measures and psychiatric evaluations. Validated PRO tools, such as Psoriatic Arthritis Impact of Disease (PsAID) [30, 31] or Routine Assessment of Patient Index Data 3 (RAPID-3) [32], can be administered to patients before or during the virtual appointment through patient gateway portals to measure symptoms from the patient’s perspective. Additionally, screening tools that incorporate subjective and/or objective assessments and assist in the early identification of PsA, such as the Psoriasis Epidemiology Screening Tool [33], can be used during virtual appointments—particularly for high-level screening of patients with suspected PsA. These assessments are useful for triaging new patients and/or evaluating therapeutic responses and disease progression in patients with established disease.

Healthcare providers should also be aware of insurance regulations regarding virtual visits, as some policies may not approve of in-person visits < 7 days after a virtual visit or allow for patient care across state lines. Providers may need to be licensed in the state the patient is receiving care during the telemedicine visit. However, a thorough virtual assessment that incorporates available tools can be useful for triaging patients until they are seen in person. Virtual appointments also enable discussions for shared decision-making between patients and providers about treatment options. If changes in treatment plans are necessary, modifications and/or medication switching can be achieved virtually using online portals that directly communicate with pharmacies. After the virtual appointment, personal health data can be transferred to patients’ electronic medical records through a virtual platform. Postvisit considerations include follow-up appointments to schedule in-person evaluations for new patients and consultations and/or referrals to specialists. While telemedicine can be useful for well-controlled follow-up appointments, provide high-level screening for PsA in new patients, and facilitate changes in treatment plans, it does present challenges for a thorough examination—especially in the evaluation of enthesitis and hidden areas of psoriatic involvement and in confirming a diagnosis of PsA. When a definitive diagnosis is needed, a telemedicine visit may allow triage for patients who need to be seen sooner when an in-person visit is delayed. In-person office visits are particularly helpful for new patients who need a thorough physical examination, imaging, or laboratory tests to ensure a comprehensive evaluation.

## High-level screening of PsA disease domains

The diagnosis and treatment of PsA can be supported by identifying and assessing 6 disease domains (psoriasis, psoriatic nail disease, dactylitis, enthesitis, peripheral arthritis, and axial disease) characteristic of PsA, which can each be evaluated via telemedicine visits or supported with telemedicine tools (Table 2). In most patients, psoriasis precedes joint disease, with a typical delay time of 7–12 years from onset to diagnosis of PsA, and psoriatic lesions can develop on the nails, which often present as pitting [34–38]. Video and/or photographic evaluations can provide essential visual information to fully assess new or changing psoriatic skin rash or plaques and psoriatic nail disease. Patients can be educated to use the validated “palm rule” to determine the BSA affected by skin involvement, or clinicians can estimate BSA by assessing the body areas affected (e.g., upper or lower limbs, trunk); however, clinicians should inquire about areas of the body not visible on video (e.g., scalp, genitals) to help provide an accurate self-assessment of skin severity due to psoriasis [23]. The definitive identification of psoriatic disease may not be possible for all presumed skin lesions, and some cases may necessitate an in-person evaluation for a differential diagnosis of psoriasis involvement.

**Table 2:** High-level screening assessments of PsA disease domains

Disease domain	Assessment tool
Psoriasis skin involvement	• Video • Photos • BSA “palm rule” • Self-administered PASI and other validated self-assessments • Directed questioning about areas of the body not visible on video (e.g., scalp, genitals)
Nail disease	• Video • Photos (e.g., pitting, onycholysis, onychomycosis)
Dactylitis	• Video • Photos • Clinician-guided examination focused on digits • Inquire about “sausage digits,” redness, warmth to touch, tenderness, and swelling
Enthesitis	• Video • Photos • Clinician-guided examination focused on commonly affected entheses • Inquire about redness and swelling at insertion sites
Peripheral arthritis	• Video (assessment of visible swelling) • Clinician-guided musculoskeletal examination focused on commonly affected joints • Inquire about swelling and tenderness at joints • Inquire about functional limitations, prolonged stiffness, and other features suggesting active inflammatory arthritis, enthesitis
Axial disease	• Video • Clinician-guided musculoskeletal examination focused on commonly affected areas of the spine; spinal mobility tests • Inquire about back pain and stiffness, particularly details on whether improved with movement or interrupts sleep • Remote scheduling and review of imaging

*BSA* body surface area; *PASI* Psoriasis Area and Severity Index; *PsA* psoriatic arthritis

Inflammatory features seen in PsA include dactylitis and enthesitis, which present as uniform swelling of entire digits and inflammation at the entheses, respectively. Dactylitis can be evaluated over a video telehealth visit and may be supported by photos. Patients can be directed to compare the suspected digit with the contralateral digit on the opposite hand or foot and to look for signs and symptoms such as redness, warmth to the touch, tenderness, and swelling [39, 40]. Clinicians should incorporate screening for visible signs of enthesitis (which can be rare), such as redness and swelling at insertion sites, and ask the patient about pain upon squeezing commonly affected entheses in virtual examinations to help assess enthesitis [11, 41]. Peripheral arthritis can result in joint pain among patients with PsA—most commonly affecting the knees, fingers, hips, ankles, and wrists [42]. Peripheral arthritis can be addressed by looking for signs of swelling and tenderness at the commonly affected joints. In a clinician-guided musculoskeletal examination, patients may be instructed to palpate their own joints or to perform functional movements on screen (such as walking, so the clinician can assess their gait to understand mobility, the ability to bear weight, and function level), which can be used to evaluate the degree of peripheral arthritis involvement [21]. Axial involvement associated with PsA can occur in 25–70% of patients, often presenting as back stiffness and pain that improves with movement [43, 44]. A high-level evaluation of back pain and stiffness through a clinician-guided physical examination (e.g., lumbar and cervical spine range of motion tests, sacroiliac joint tests, occiput palpation) [21, 22] and patient-reported questionnaires that address mechanical vs. inflammatory back pain can be conducted during telehealth screening to assess the management of axial disease in patients with PsA. Additionally, clinicians can remotely schedule imaging appointments for patients if needed to better evaluate axial involvement and can virtually review imaging scans if off-site imaging facilities are used. These clinical characteristics can be challenging to evaluate when patients have concurrent symptoms [11, 36]; in such instances, clinician training and screening tools can help with PsA diagnosis and management.

## Screening tools for PsA that can be used to triage patients for referrals

Validated screening tools, such as the Psoriasis Epidemiology Screening Tool [33], have been developed for routine clinical practice and can be applied for telemedicine triage of new patients alongside a thorough patient history to assist in the early detection and identification of PsA in the absence of a face-to-face visit. It is imperative to collect a comprehensive patient history to apply to screening tools and to incorporate risk factors associated with PsA, such as family history. Patients with a family history of PsA have been reported to be 27 to 48 times more likely to develop PsA than controls [45–47]. Screening tools have strengths and weaknesses but can be used to facilitate the early diagnosis of PsA (Table 3) [48, 49].

**Table 3:** Screening tools for the diagnosis of PsA and assessments for quality of life, functional ability, and disease activity measures to evaluate the disease impact

Screening tool	Description
PEST	• Self-assessment tool of 5 questions about symptoms and history and a sketch of a mannequin for patients to highlight which joints are affected • Convenient, fast to complete, and available for free • Cutoff for PsA is a score of 3 • Specificity of 78% and high sensitivity (92%) • PEST was reported to be favorable to EARP and PASE in the trade-off between sensitivity and specificity to detect PsA
PSA	• **P**ain (joints), **S**tiffness/**S**ausage digit (> 30 min of stiffness after a period of inactivity and/or presence of dactylitis “sausage digit”), and **A**xial (axial spine involvement, back pain associated with stiffness and pain that improves with activity) • Teaching tool developed for the recognition of early PsA • Convenient and helpful for a rapid assessment in routine clinical settings
PRO measure	
RAPID-3	• RAPID-3 includes an assessment of physical function and a PtGA for pain and for global health - Score cutoff recommendations for RAPID-3 range from 0–3.0 for remission and 3.1–6.0 for low, 6.1–12.0 for moderate, and 12.1–30 for high severity • RAPID-3 is available online: https://www.rheumatology.org/Portals/0/Files/RAPID3%20Form.pdf
PsAID questionnaires	• PsAID questionnaires are used to evaluate the impact of PsA on patients’ lives that can be grouped into 3 categories based on impact: physical, related to skin, and psychological and social • PsAID-9 is the short version for use in clinical trials - PASS cutoff for PsAID-9 score is 4 with a 3.6-point change • PsAID-9 is available online: http://pitie-salpetriere.aphp.fr/psaid/raid_psaid_quest_home.php • The PsAID-12 long version for use in clinical practice - PASS cutoff for PsAID-12 score is 4 with a 3.0-point change - Provisionally endorsed as a core PRO measure for health-related QoL in PsA clinical trials • PsAID-12 is available online: http://pitie-salpetriere.aphp.fr/psaid/raid_psaid_quest_home.php
HAQ-DI	• HAQ-DI is composed of 20 questions in 8 categories that encompass a comprehensive set of routine activities to assess functional ability • The scores from each category are averaged into an overall HAQ-DI score (0–3) • HAQ-DI is available online: http://integrationacademy.ahrqdemo.org/sites/default/files/HAQ-DI_0.pdf

*EARP* Early Arthritis for Psoriatic Patients; *HAQ-DI* Health Assessment Questionnaire Disability Index; *PASE* Psoriatic Arthritis Screening and Evaluation; *PASS* patient-acceptable symptom state; *PEST* Psoriasis Epidemiology Screening Tool; *PRO* patient-reported outcome; *PsA* psoriatic arthritis; *PsAID* Psoriatic Arthritis Impact of Disease; *PtGA* patient global assessment; *QoL* quality of life; *RAPID-3* Routine Assessment of Patient Index Data 3

Although not validated as a screening tool, the mnemonic **PSA**—which represents **P**ain (joints), **S**tiffness/**S**ausage digit (> 30 min of stiffness after a period of inactivity and/or presence of dactylitis, “sausage digit”), and **A**xial (axial spine involvement, back pain associated with stiffness and pain that improves with activity)—was developed to help identify the early characteristic features of PsA, particularly for use by general clinicians as a means to identify patients who should be referred to a rheumatologist [11, 48]. This mnemonic is a straightforward way to enhance awareness of PsA, facilitate rapid screening, and emphasize the core features of the disease; patients with psoriasis who have ≥ 2 of the PSA mnemonic items are more likely to develop PsA.

Screening tools, especially those with images, provide direction and instruction to help patients understand clinician inquiries, help prevent missed information, and remind clinicians about the proper questions to ask to distinguish PsA. The increased use of proper diagnostic criteria, training to identify distinguishing characteristics of PsA, and screening tools are necessary to decrease the frequency of undiagnosed PsA.

## Application of key measures of disease burden in virtual appointments

Disease burden in patients with PsA that is caused by clinical manifestations of the disease can result in significantly poorer QoL compared with the general population [50–54] and similar QoL impairments as patients who have more severe peripheral joint disease activity or other severe rheumatic diseases [55, 56]. All domains are important for the overall assessment of PsA, and the value of understanding the relationship between joint and skin components and their relative contribution to disease burden is imperative to the evaluation of disease progression and management [57, 58]. A myriad of tools for assessing QoL, functional ability, and disease activity are available for PsA and can be applied during telemedicine visits to gauge a patient’s status and therapeutic response [59, 60].

QoL indices that comprise multiple measures of PsA and other rheumatic diseases include the RAPID-3 and PsAID-12/PsAID-9 questionnaires (Table 3) [30, 32, 61–64]. RAPID-3 is a validated tool to quickly assess patient-reported physical function, pain, and patient global estimate of status [32]. It is responsive to treatments for PsA and has been correlated with measures of disease activity, such as the Disease Activity in Psoriatic Arthritis, Psoriatic Arthritis Disease Activity Score, Disease Activity Score in 28 joints, and Clinical Disease Activity Index [62, 65–68]. PsAID-9 and PsAID-12 questionnaires were developed by a European League Against Rheumatism task force to provide tools that could assess the impact of PsA from the perspective of the patient [30]. PsAID-12 is used in routine clinical practice and the PsAID-9, a shortened version of PsAID-12, is used primarily in clinical trials [69]. Each component represents a PsA-specific life-impact domain that can assist healthcare providers in making shared decisions with patients about treatment plans. PsAID-9/PsAID-12 scores ≤ 4 represent the **P**atient-**A**cceptable **S**ymptom **S**tate (PASS); scores > 4 may necessitate a change in treatment plan [30]. PsAID-12 scores have been shown to be significantly correlated with other measures of PsA disease activity and are responsive to treatment [30, 50, 56, 61, 70–72]. RAPID-3 and PsAID-12 have shown construct validity and are suitable for use in routine clinical settings for patients with PsA [73]. These PRO tools are practical for the continued assessment of the impact of disease and therapeutic response in the absence of in-person visits and are accessible online for patients and clinicians.

Questionnaires that assess functional impairment and disease activity can also be used to gauge disease status, progression, and therapeutic response during previsit screenings and follow-up telehealth visits. The Health Assessment Questionnaire Disability Index has been established as an instrument to measure functional disability resulting from disease progression and/or severity [64, 74]. Questions that address fine and locomotor movements of both the upper and lower extremities are included to encompass a comprehensive set of routine functional activities [63]. The Health Assessment Questionnaire Disability Index has been significantly associated with clinical therapeutic response and radiographic progression in patients [75–77]. Online administration of accessible QoL and functional instruments allows for increased frequency of evaluation of disease impact and can provide the opportunity for rapid review, shared decision-making, and easier identification of the need for treatment changes.

## Resources and recommendations for the use of telemedicine in routine clinical care

As telemedicine becomes more commonplace, it is important to emphasize healthcare access and maintain treatment regimens for patients with PsA. The use of telemedicine is not a new practice; it has been used in many countries for years to close the gap in access to specialty care in remote and rural areas, with both reported accuracy and satisfaction among patients and clinicians [78, 79]. Reports of patient satisfaction and the accuracy of video consultations support the potential for telemedicine to assist in the high-level screening of patients with suspected PsA and to maintain routine appointments for patients with established disease. Key concerns raised by clinicians about the application of telemedicine include issues related to implementation (particularly for individual practices), payment for services provided, and liability [20]. Several resources from professional organizations and government offices have been created to help providers and support staff navigate the changes required for telemedicine initiation and the challenges that may arise (Table 4).

**Table 4:** Guidelines and resources for rheumatologists and dermatologists for best telemedicine practices

COVID-19 practice and advocacy resources (ACR)	The ACR compiled resources for providers containing detailed information: • Fact sheet with coding and practical guidelines • List of temporary changes made by commercial payers to their telemedicine policies • Quick coding references guides • List of telemedicine platforms available for use by rheumatology practices • A chart with details on malpractice coverage for telemedicine services The ACR also compiled information for patients on telemedicine use during COVID-19 Available online: https://www.rheumatology.org/Announcements/COVID-19-Practice-and-Advocacy
Telemedicine quick guide and implementation playbook (AMA)	The AMA compiled resources to support clinicians and practices to expedite the implementation of telemedicine: Quick guide: https://www.ama-assn.org/practice-management/digital/ama-telehealth-quick-guide Implementation playbook: https://www.ama-assn.org/system/files/2020-04/ama-telehealth-playbook.pdf
Telemedicine payment policies (AMA)	The AMA compiled a list of COVID-19 telemedicine payment policies comparing insurance providers Available online: https://www.ama-assn.org/system/files/2020-09/covid-19-telehealth-payment-policies.pdf
Quick-start guide to telehealth during a health crisis (ATA)	The ATA developed a detailed guide to help healthcare providers initiate telemedicine during a health crisis Available online: https://info.americantelemed.org/covid-19-resources-quickstart-guide-landing
HIPAA rules and enforcement (OCR)	The OCR provides a list of HIPAA-compliant vendors and updates the enforcement of HIPAA requirements Available online: https://www.hhs.gov/hipaa/for-professionals/special-topics/emergency-preparedness/notification-enforcement-discretion-telehealth/index.html
List of executive orders for licensure (FSMB)	The FSMB tracks current executive orders related to state licensure Available online: https://www.fsmb.org/advocacy/covid-19/
Teledermatology toolkit (AAD)	The AAD compiled a toolkit for healthcare providers to navigate the use of telemedicine Available online: https://www.aad.org/member/practice/telederm/toolkit
How to prepare for a telemedicine appointment (AAD)	The AAD put together simple steps to help patients prepare for telemedicine appointments Available online: https://www.aad.org/public/fad/telemedicine/telemedicine-prepare-appointment
Guidance for patients during COVID-19 outbreak (EULAR)	EULAR provided treatment guidance for patients who are treated with immunosuppressive drugs, including DMARDs Available online: https://www.eular.org/eular_guidance_for_patients_covid19_outbreak.cfm
Telemedicine resources for providers and patients (HRSA)	HRSA of HHS provided information about the latest information and resources for general telemedicine policies and procedures Available online: https://www.telehealth.hhs.gov/
Healthcare provider fact sheet for Medicare telemedicine (CMS)	Summary of changes to Medicare coverage and payment of virtual services Available online: https://www.cms.gov/newsroom/fact-sheets/medicare-telemedicine-health-care-provider-fact-sheet
How to prepare for a rheumatology telemedicine visit during the coronavirus pandemic (CreakyJoints)	CreakyJoints put together patient-focused information to help patients prepare for telemedicine appointments Available online: https://creakyjoints.org/living-with-arthritis/coronavirus/daily-living/tips-for-telehealth-rheumatology-visit/

*AAD* American Academy of Dermatology; *ACR* American College of Rheumatology; *AMA* American Medical Association; *ATA* American Telemedicine Association; *CMS* Centers for Medicare and Medicaid Services; *DMARD* disease-modifying antirheumatic drug; *EULAR* European League Against Rheumatism; *FSMB* Federation of State Medical Boards; *HHS* US Department of Health and Human Services; *HIPAA* Health Insurance Portability and Accountability Act; *HRSA* Health Resources and Services Administration; *OCR* Office of Civil Rights

The American College of Rheumatology supports the use of telemedicine for appropriate patients, particularly during the COVID-19 pandemic, and defers to rheumatologists and rheumatology healthcare professionals to determine what defines routine, urgent, and emergent care for patients [80].

The AMA and the American Telemedicine Association (ATA) have developed an implementation playbook and a quick-start guide to telehealth, respectively, to provide a comprehensive introduction regarding telemedicine and practical tools to apply for its implementation into clinical practice [20, 81]. The AMA endorses the use of telemedicine and remote care services to ensure the safe management of continued care during the COVID-19 pandemic and for patients in remote areas outside the pandemic setting. The AMA and ATA provide recommendations and guidelines to efficiently set up telemedicine in clinical practice and include resources for healthcare providers to stay current and knowledgeable about telemedicine [20, 81]. Protocols that define when a telemedicine visit is appropriate and how to best conduct a virtual appointment should be developed before implementing telemedicine in a medical practice. Patient education and engagement are important to ensure the success of telemedicine visits, and the AMA and ATA recommend proactive patient outreach and the development of a plan to best support patients on how to access and use telemedicine technology. The associations also outline tips to identify the appropriate platform for optimal care, discuss financial considerations, and provide training guides for support staff.

The rules, regulations, and financial aspects of telemedicine are evolving with the increased use of telemedicine services, so it is essential to be aware of the most current information that may impact clinical practices or organizations. Several resources providing rules and regulations on telemedicine policies and guidelines concerning medical coding, reimbursement, and other relevant issues are available, which may vary by state (Table 4). During the pandemic, some states temporarily waived licensure requirements to expand interstate access to healthcare, allowing patients to see out-of-state providers virtually [82]. Recently, all 50 states in the USA and the District of Columbia allowed for the reimbursement of some live video telemedicine services; however, the reimbursement policies vary among states [83]. Coverage policies also vary by state and insurer type; however, both private insurance and Medicaid/Medicare have expanded their coverage to include telemedicine during the pandemic. The Centers for Medicare and Medicaid Services and individual private insurance policies can be used to verify updated telemedicine coverage. The OCR of the HHS relaxed some HIPAA and state-related barriers and the enforcement of some penalties, as long as clinicians act in the best interest of patients.

Many of the recent changes in policy concerning the use of telemedicine are tied to the ongoing pandemic and may be temporary; therefore, long-term considerations are needed to establish telemedicine that is sustainable in clinical practice.

## The future of telemedicine in dermatology and rheumatology

Lessons can be learned from the advances in telemedicine during the pandemic and applied for the development of telemedicine services in rheumatology and dermatology in the future. The ability to deviate from a set office schedule can offer healthcare providers increased autonomy and flexibility concerning patient care, which can potentially diminish provider burnout. Telemedicine also may alleviate extensive provider shortages, which can affect urban and rural communities across many specialties [84]. The projected demand for adult rheumatology providers is estimated to greatly exceed—by up to 102%—the clinical full-time practitioner workforce by 2030, which stresses the need for innovative strategies to manage access to healthcare, such as telemedicine [85].

The introduction of modern technology and tools into routine clinical practice can help advance the use of telemedicine, overcome obstacles to geographic and provider availability, and increase patient engagement in research and disease management [86]. A combination of mobile health devices and telemedicine visits to facilitate communication and data input have been applied in virtual clinical trials with success [87–89] and have been incorporated in planned initiatives to address gaps in PsA research [27, 28]. Substantial reductions in the cost and time to complete clinical trials can be achieved with a shift to virtual methods, which can allow for the centralization of data collection and a decrease in the resources needed to manage participating sites. Technologies available for rheumatologic and/or dermatologic clinical trials include social media outlets for recruitment, online surveys for collection of patient-reported data, virtual study appointments, and online portals for communication between patients and clinical staff [89]. More recently, the incorporation of wearable sensors and handheld exam kits, such as mobile electronic dolorimeters and stethoscopes, into clinical trials and routine clinical practice have increased the options for objective outcome measures that can be easily assessed with accuracy in the absence of a trained clinical provider.

Barriers to the application of telemedicine, such as poor internet connectivity, lack of familiarity and discomfort with the use of technology, and limited access to high-quality tools, can be attributed to multiple factors, including patient circumstances and demographics. Limited experience with technology can make it difficult for some patients to access electronic medical record patient portals and to establish optimal conditions to capture quality images and videos. Incorporation of telemedicine into a clinical practice increases the burden to providers and clinical teams, e.g., increased costs associated with training and instruments needed to adapt to virtual care. Additionally, providers will need to stay current on the laws and regulations regarding patient care via telemedicine. Furthermore, patients and providers need to feel confident that these tools meet standards for quality care; otherwise, in-person visits may be preferable. Virtual appointments have some disadvantages: less-personal patient-provider interaction, patient discomfort with being examined virtually, limited ability of the provider to inspect areas of the body via images and/or video, and inability of the provider to conduct certain diagnostic tests. For a complex disease such as PsA, an in-person physical examination may be difficult to replace due to many of these challenges, particularly for new patients who require a definitive diagnosis. A standardized virtual evaluation may be required to ensure a differential PsA diagnosis to prevent treatment delay. Further studies to validate the use of telemedicine tools and instruments, and to increase education and awareness about the capabilities of virtual care, are needed to address these challenges so that telemedicine can be used properly and increasingly incorporated into routine clinical care.

The increased incorporation of telemedicine services into clinical practice presents an opportunity for providers to optimize resources and improve patient outcomes. Telemedicine has the potential to increase access to care by rheumatologists and dermatologists, provide opportunities for training and education regarding PsA, save costs, and decrease travel time attributed to office visits.

## Summary

PsA is difficult to distinguish from other rheumatic diseases and is commonly misdiagnosed and/or undiagnosed, especially in patients without psoriasis. It is essential that clinicians apply proper screening tools, ask pointed questions to properly evaluate patient symptoms, and use appropriate PRO assessments with telemedicine services. Application of the guidelines and resources described herein has the potential to result in the optimal use of telemedicine in the diagnosis of PsA.

## Data Availability

Not applicable to this manuscript.

## Code availability

Not applicable to this manuscript.
